# The Sound of Feelings: Electrophysiological Responses to Emotional Speech in Alexithymia

**DOI:** 10.1371/journal.pone.0036951

**Published:** 2012-05-15

**Authors:** Katharina Sophia Goerlich, André Aleman, Sander Martens

**Affiliations:** 1 Neuroimaging Center, Department of Neuroscience, University Medical Center Groningen, University of Groningen, Groningen, The Netherlands; 2 Department of Psychology, University of Groningen, Groningen, The Netherlands; University of Florida, United States of America

## Abstract

**Background:**

Alexithymia is a personality trait characterized by difficulties in the cognitive processing of emotions (cognitive dimension) and in the experience of emotions (affective dimension). Previous research focused mainly on visual emotional processing in the cognitive alexithymia dimension. We investigated the impact of both alexithymia dimensions on electrophysiological responses to emotional speech in 60 female subjects.

**Methodology:**

During unattended processing, subjects watched a movie while an emotional prosody oddball paradigm was presented in the background. During attended processing, subjects detected deviants in emotional prosody. The cognitive alexithymia dimension was associated with a left-hemisphere bias during early stages of unattended emotional speech processing, and with generally reduced amplitudes of the late P3 component during attended processing. In contrast, the affective dimension did not modulate unattended emotional prosody perception, but was associated with reduced P3 amplitudes during attended processing particularly to emotional prosody spoken in high intensity.

**Conclusions:**

Our results provide evidence for a dissociable impact of the two alexithymia dimensions on electrophysiological responses during the attended and unattended processing of emotional prosody. The observed electrophysiological modulations are indicative of a reduced sensitivity to the emotional qualities of speech, which may be a contributing factor to problems in interpersonal communication associated with alexithymia.

## Introduction

Alexithymia is a personality trait characterized by difficulties in the cognitive processing and experience of emotions. With a prevalence rate of up to 10 percent [1], alexithymia has been recognized as a risk factor for a variety of psychiatric and medical disorders, including somatization, anxiety, depression, hypertension, and chronic pain [2]. In addition, alexithymia exhibits high comorbidity with disorders of the Autism spectrum [3]–[6].

The term alexithymia (‘no words for feelings’) was coined by Sifneos [7] to describe individuals who exhibited difficulty identifying, analyzing, and verbalizing their feelings. In addition to these cognitive impairments in emotional processing (cognitive dimension), alexithymia is defined by difficulty emotionalizing (the degree to which someone is emotionally aroused by emotion-inducing events) and fantasizing (the degree to which someone is inclined to imagine, day-dream, etc.). These latter two characteristics refer to the level of emotional experience (affective dimension). While the majority of research on alexithymia has focused on its cognitive dimension, the importance of its affective dimension has recently been pointed out [8], and the two dimensions have been suggested to exert a dissociable impact on emotional processing [9], [10].

Individuals with alexithymia show a paucity of facial emotional expressions and a somewhat stiff wooden posture [2], are described as cold and distant [11] and interpersonally indifferent [12], leading to problems in social communication. Behavioral studies demonstrated that alexithymia is associated with impairment identifying facial expressions of emotion [13]–[15] matching verbal with non-verbal emotional stimuli [16], and remembering words with emotional connotations [17].

### Neurobiological dysfunction in alexithymia

The right hemisphere is thought to be more involved in emotion processing than the left hemisphere [18], [19], a model based on evidence for verbal, analytical, conscious processing taking place in the left hemisphere [20], [21] and nonverbal, emotional, subconscious processing taking place in the right hemisphere [22]–[25]. Derived from this model, alexithymia has been proposed to result from a deficit in the interhemispheric communication between the two cerebral hemispheres, or from a dysfunction of the right hemisphere, possibly paired with a left hemisphere preference for the processing of emotions [26], [27]. Several studies have provided evidence for a hypoactivity of the right hemisphere and a hyperactivity of the left hemisphere in alexithymia. For instance, right as compared to left hemisphere stroke patients showed a higher prevalence of alexithymia [28]. In a positron-emission tomography (PET) study, Kano and colleagues [29] observed lower regional cerebral blood flow (rCBF) during the viewing of emotional faces in a distributed right-hemispheric network in high-versus low-scorers on alexithymia. Furthermore, Jessimer and Markham [30] studied the ability of high- and low-scorers on alexithymia to attribute emotional value to chimeric pictures of faces composed of conjoined emotive and nonemotive halves. Normally, right-handed individuals tend to choose the chimeric face with the emotive half on the left as being more expressive than the half on the right, indicating a leftward bias related to a predominantly right-hemispheric processing of these stimuli [31]. In contrast, high-scorers on alexithymia showed significantly less left bias on chimeric tasks than low-scorers, indirectly suggesting less right-hemispheric involvement [30]. Using a lateralized visual-matching task, Bermond and coworkers [32] demonstrated that high-scorers on alexithymia as compared to low-scorers showed a left hemisphere preference for the processing of emotional words. Finally, a dysfunction of the right hemisphere during emotional processing in alexithymia has been suggested by studies using electroencephalography (EEG) [33], [34].

### The electrophysiology of emotion processing in alexithymia

Electrophysiology with its extremely high temporal resolution in the range of milliseconds is an excellent means to investigate how emotional processing unfolds in time and can give information about whether alexithymia primarily affects overt, appraisal-related aspects of emotional processing or whether it affects already the more automatic, early perceptual-related aspects. The findings of studies employing event-related potentials (ERPs) to address this question will be summarized below. However, it should be kept in mind that previous ERP studies mostly relied on the 20-item Toronto Alexithymia Scale (TAS-20) to assess levels of alexithymia. This scale assesses only the cognitive alexithymia dimension, i.e. difficulty identifying, analyzing, and verbalizing feelings. Therefore, previous findings of ERP studies on alexithymia primarily refer to cognitive deficits in emotional processing, whereas the impact of disturbances in emotional experience (affective alexithymia dimension) has remained elusive.

Franz et al. [35] presented high- and low-scorers on the TAS-20 alexithymia scale with aversive versus neutral pictures and observed that high-scorers on alexithymia exhibited elevated amplitudes of the P2 component in response to aversive pictures. The authors interpreted this finding to reflect higher effort and recruitment of additional cognitive resources to process emotional stimuli in individuals with alexithymia. Bermond et al. [36] assessed alexithymia using the Bermond-Vorst Alexithymia Questionnaire (BVAQ) [8], which covers both the cognitive and the affective alexithymia dimension, and divided participants into two groups with either high or low scores on the sum score of both dimensions. The authors reported reduced P3 amplitudes during negative picture processing in female, but not male high-scorers on alexithymia, compared to low-scorers. No impact of alexithymia on latencies of the P3 was observed. Using morphed angry and disgusted facial expressions in an emotion categorization task, Vermeulen et al. [37] specifically focused on ERP latencies. Latencies of the P3 did not differ as a function of alexithymia, but the N2b/P3a complex showed delayed latencies in high-scorers on the TAS-20 scale as compared to low-scorers, indicating an overall delayed categorical perception of emotional faces in alexithymia.

Pollatos and Gramann [38] investigated early electrophysiological responses to emotional pictures in order to test whether early processing deficits contribute to deficits at later processing stages in alexithymia. The authors observed that amplitudes of the early component P1 were reduced in high-scorers on the TAS-20 scale during the processing of positive and neutral pictures, predicted by the alexithymia subscale “difficulty describing feelings”. The same subscale predicted larger amplitudes of the N2 for negative and neutral pictures in high-scorers. In line with Bermond and colleagues [36], amplitudes of the later occurring P3 were reduced at posterior regions in response to negative pictures in high-scorers on alexithymia. Further, P1 amplitudes were found to co-vary with P3 amplitudes, indicating that early processing deficits might indeed contribute to deficits during later emotional processing in alexithymia.

Confirming the observation of an impact of alexithymia on both early and late electrophysiological processing of emotions, Walker and colleagues [39] found reduced N2 and larger P2 amplitudes during the suppression of emotion elicited by negative images in low–scorers, but not high-scorers on the TAS-20 alexithymia scale. Further, they identified reduced amplitudes of the late positive potential (LPP) in a time-window of 400–600 ms post picture onset with increasing scores on alexithymia during negative emotion suppression, suggesting that alexithymia was inversely related to the magnitude of emotion-related ERP activity during emotion suppression. This alteration of late positive potentials during emotion regulation was confirmed by Pollatos and Gramann [40], who reported reductions in amplitudes of the P3 and the slow wave in the course of successful cognitive reappraisal of negative emotion only in individuals with low scores, but not in those with high scores on alexithymia.

Taken together, ERP studies investigating visual emotional processing suggested that alexithymia influences both early (<300 ms) and late (>300 ms) emotional processing. At late processing stages, thought to reflect cognitive appraisal of emotion, there is converging evidence for reduced emotional processing as a function of alexithymia as reflected in diminished amplitudes of the later occurring P3 component [36,38, but see 37] and the LPP [39] as well as in a failure to down-regulate P3 and slow wave amplitudes through reappraisal of negative emotion [40]. Findings of differences in early components during emotion processing in relation to alexithymia, thought to reflect more automatic, perceptual processing, are less consistent with respect to directionality as both increased [35], [38] and decreased [38] amplitudes of early ERP components have been reported.

### Alexithymia and emotional prosody

In contrast to the visual domain, auditory emotional processing has rarely been investigated in relation to alexithymia. A recent behavioral study tested the impact of emotional background music on the recognition of emotion words [41]. Exposure to angry music was found to result in decreased recognition rates of emotional words in high-scorers as compared to low-scorers on alexithymia (cognitive dimension). In an ERP study, Schäfer and colleagues [42] presented alexithymic versus non-alexithymic participants with aversive white noise. They identified significantly larger amplitudes of the P1-N1 complex (40–200 ms post stimulus onset) in alexithymics compared to non-alexithymics in response to aversive white noise, while intensity and pleasantness of the aversive stimuli were rated equally by the two groups. These results were interpreted as indicative of a hypersensitivity to unpleasant external stimulation and provide further evidence for a modulation of early ERP components by alexithymia.

Emotional prosody, the ‘melody of speech’, is an important means to understand the emotional state and intention of others in social communication. A recent meta-analysis showed that emotional prosody perception, though processed by both hemispheres, is relatively lateralized to the right hemisphere [43]. How alexithymia affects the processing of the emotional qualities of speech has only been investigated by two previous studies. In a behavioral study, Swart and coworkers [15] presented high- and low-scorers on the verbalizing subscale of the BVAQ with sentences conveying an emotional content (e.g., sad) spoken in incongruous (e.g., happy) emotional prosody. No statistically significant differences in emotional prosody identification were observed as a function of alexithymia. Nevertheless, it is conceivable that alexithymia affects emotional prosody comprehension in a more subtle manner evading detection through behavioral measures. ERPs with their measurement sensitivity in the range of milliseconds are potentially more suited to detect such subtle processing impairments. Following this rationale, we conducted a previous ERP study [44] using emotional prosody, music, and words with emotional connotations in order to test the impact of TAS-20 alexithymia scores on cross-modal affective priming as well as on amplitudes of the N400, an indicator of the perception of mismatches in affective meaning [45]. In line with Swart and colleagues, no behavioral differences were observed. However, alexithymia correlated negatively with N400 amplitudes during affective categorization of happy and sad prosody and music targets, confirming our hypothesis of a reduced sensitivity during the perception of mismatches in the emotional qualities of speech and music with increasing alexithymia scores [44].

The present study was designed to further investigate the impact of alexithymia on the electrophysiological processing of emotional speech, taking its two dimensions into account. In addition to the attended processing of emotional speech (participants detected deviants in emotional prosody), unattended emotional speech processing was tested (participants watched a movie while emotional prosodic stimuli were played in the background). An auditory oddball paradigm was employed in both tasks, in which occasional deviant stimuli (20%) were presented in a sequence of frequent standard stimuli (80%). The relation between alexithymia and abilities to identify emotions conveyed by speech was further tested in a behavioral (off-line) task.

### Emotional prosody and event-related potentials

ERP components of interest during attended emotional prosody processing (deviant detection) are the early components N1 and P2 as well as the late component P3 [46]. The N1 is a negative deflection with a central maximum peaking 100 ms after the onset of a prosodic stimulus. It is generated in bilateral secondary auditory cortex [47] and reflects the extraction of acoustic cues (e.g., stimulus frequency and intensity) during early acoustic processing. The amplitude of the N1 increases with the amount of attention devoted to an acoustic stimulus [48], [49]. The P2 is a positive deflection occurring 200 ms after stimulus onset with an anterior maximum. It is thought to reflect the initial detection of emotional salience in auditory material (i.e., early emotional appraisal) independent of whether the stimuli contain semantic emotional information [50], [51]. The P3 is a longer-lasting later occurring positivity with a centroparietal maximum starting at 300 ms after the prosodic stimulus. It reflects the cognitive evaluation and classification of task-relevant targets and is therefore related to the decisional, response-related processing stage. The P3 is highly dependent on stimulus context and levels of attention and arousal [52]. Reduced amplitudes and prolonged latencies of the P3 are often used as indicators of cognitive impairment in psychopathology [53], reflecting reduced cognitive resource allocation to task-relevant stimuli and a slowing down of cognitive processes.

The ERP component of interest during early unattended processing of emotional prosody (movie watching with prosodic stimuli played in the background) is the Mismatch Negativity (MMN). The MMN, elicited without the participant's attention, occurs between 100 and 200 ms after the onset of a prosodic stimulus and is generated in secondary auditory cortex and inferior frontal cortex [54]. It reflects the formation of memory traces and the detection of differences between auditory stimuli [55], [56], and its amplitude varies with the amount of personal significance assigned to the deviating event. The MMN is thought to reflect higher-order perceptual processes underlying stimulus discrimination rather than only the encoding of simple physical differences between stimuli [57]. Complex stimuli may elicited an MMN with two peaks, with the early peak (eMMN) reflecting the detection of differences based on acoustic stimulus features and the later peak (lMMN), sometimes termed ‘late discriminative negativity’ reflecting higher-order integrative processes in auditory perception and a more global, ‘gestalt-based’ processing of auditory stimuli [58], [59].

Early components (<300 ms) such as the N1 and the P2 have been suggested before to differ as a function of alexithymia by studies on visual emotional processing [35], [38] and by a study on the processing of aversive auditory information [42]. Later components (>300 ms) such as the P3, LPP, and N400 have been found to be reduced during visual [36], [38], [39] and visual-auditory emotional processing in alexithymia [44]. Though not investigated in relation to alexithymia, the MMN has been reported to differ in individuals with Asperger's syndrome [59], [60], part of the Autism Spectrum with which alexithymia exhibits high comorbidity [3], [4].

In light of the existing evidence, we hypothesized a modulation of the early N1 and P2 components by alexithymia during the detection of deviants in emotional prosody, as well as of the MMN during unattended emotional speech processing. We further predicted a reduced sensitivity during overt processing of emotional speech qualities, reflected in reduced amplitudes of the P3 with increasing alexithymia scores. In addition, we hypothesized to find evidence for a left hemisphere preference during the perception of emotional speech with increasing alexithymia scores. Lastly, we wished to determine whether the cognitive and the affective alexithymia dimensions exert a dissociable impact on the attended and unattended processing of emotional prosody and on the ability to identify emotions conveyed by speech.

A purely female sample of participants was chosen taking into account previously demonstrated gender differences in emotional prosody perception at the behavioral as well as at the electrophysiological level [61]–[66]. For instance, in an oddball paradigm using emotional and neutral prosody, larger amplitudes of the mismatch negativity (MMN), an event-related potential (ERP) also used in the present study occurred in female, but not in male participants during unattended perception of deviants in emotional prosody [64].

## Methods

### Participants

The 20-item Toronto Alexithymia Scale (TAS-20) was used as a brief assessment tool of alexithymia scores in a total sample of 1039 female students from the University of Groningen. From this total sample, twenty students with high TAS-20 scores (57–72, mean 62.9, SD 4.7) and 20 students with low TAS-20 scores (20–35, mean 28.9, SD 3.6) were selected and invited to the EEG session, along with 20 students with average scores on the TAS-20 (40–48, mean 43.7, SD 2.7). Extreme (low and high) scorers on the TAS-20 scale were chosen in line with previous studies [26], [27]. Individuals with average scores were additionally included in order to ensure a broad and continuous spectrum of alexithymia scores on the cognitive dimension of the Bermond-Vorst Alexithymia Questionnaire (BVAQ), which correlates to approx. 80% with the TAS-20 [8], and to increase the probability of a wide range of scores on the affective alexithymia dimension, which the BVAQ assesses in addition. Scores on the affective and the cognitive dimension of the BVAQ, which was to be filled out directly after the EEG session were then used in correlation analyses to test whether the two alexithymia dimensions show a differential impact on the attended and unattended processing of emotional prosody.

All participants were healthy female native speakers of Dutch (age range 18–25 years), with no neurological or psychiatric disorders in present or past, normal hearing, and normal or corrected-to-normal vision. Participants received € 20 for their participation in the study.

### Ethics statement

The Medical Ethical Committee of the University Medical Center Groningen approved the experimental protocol, and all participants gave written informed consent prior to the study. The study was conducted in accordance with the Declaration of Helsinki.

### Toronto Alexithymia Scale (TAS-20)

The TAS-20 is the most widely used measure of alexithymia with a demonstrated validity, reliability, and stability [67], [68]. A validated Dutch translation of the scale was used for the present study. The scale consists of 20 self-report items rated on a 5-point Likert scale (1: strongly disagree, 5: strongly agree), with five negatively keyed items.

The TAS-20 comprises three subscales assessing alexithymia at a cognitive level: (1) difficulty identifying feelings (e.g., “I often don't know why I'm angry”), (2) difficulty describing feelings (e.g., “I find it hard to describe how I feel about people”), and (3) externally oriented thinking (e.g., “I prefer talking to people about their daily activities rather than their feelings”). Possible scores range from 20 to 100, higher scores indicate higher degrees of alexithymia.

### Bermond-Vorst Alexithymia Questionnaire (BVAQ)

The BVAQ is a 40-item self-report scale, which consists of five subscales with eight items per scale [8]. The five subscales are: 1) (Difficulty) Verbalizing one's own emotional states, (2) (Difficulty) Identifying the nature of one's own emotions, (3) (Difficulty) Analyzing one's own emotional states, (4) (Difficulty) Fantasizing: the degree to which someone is inclined to imagine, day-dream, etc., and (5) (Difficulty) Emotionalizing: the degree to which someone is emotionally aroused by emotion-inducing events. Answers are scored on a 5-point Likert scale (1 = certainly does not apply to me, 5 = certainly applies to me).

The five-subscale structure of the BVAQ corresponds to the original description of alexithymia by Nemiah and Sifneos [7], [69], who had defined the alexithymia concept by the following features: reduced capacities in emotionalizing, fantasizing, identifying emotions, verbalizing emotions, and pensé opératoire (externally oriented thinking) or analyzing emotions. The three subscales identifying, analyzing, and verbalizing feelings assess the cognitive alexithymia dimension. There is substantial overlap between the cognitive subscales of the BVAQ and the TAS-20, reflected in a high correlation between the sum scores on these three BVAQ subscales and the TAS-20 sum score (*r* = .80) [8], [70], indicating that these scales measure the same features [8]. The two BVAQ subscales emotionalizing and fantasizing assess the affective dimension of alexithymia. The validity of this two-factor structure of the BVAQ with an affective versus a cognitive alexithymia dimension has been demonstrated by factor-analyses in six languages and seven populations [71]–[73]. High scores on the cognitive alexithymia dimension indicate low abilities to identify, analyze, and verbalize feelings. High scores on the affective alexithymia dimension indicate low abilities to emotionalize and fantasize.

### Materials

An auditory oddball paradigm with 80% standards and 20% deviants was created for the present study. Nonsense syllables (baba, dada, gaga) spoken in neutral, happy, angry, sad, and disgusted intonation in low (e.g., a bit sad) and high (e.g., very angry) intensity constituted the stimuli of this paradigm. Nonsense syllables were chosen in order to exclude interference by semantic processing, enabling us to specifically measure electrophysiological responses to variations in emotional prosody. The syllables “baba”, “dada”, “gaga” were chosen because they have the same consonant (C) – vowel (V) structure (CVCV), employ the same vowel and contain only voiced consonants, keeping acoustic features of the stimuli constant across conditions.

The stimuli were recorded with the help of a semiprofessional actress, who pronounced the syllables in neutral, happy, angry, sad, and disgusted prosody with low and high emotional intensity. The recorded stimuli were cut to a length of approximately 600 ms and amplitude normalized using the Praat speech processing software [74]. The procedure amplified every stimulus item such that the digitalized sample with the maximum amplitude was set at the maximum positive or negative value of the converter range, and all other samples were scaled proportionally. As a result, all stimuli had about equal volume.

The stimuli were validated in two pilot studies with 13 independent raters each. The raters were asked to indicate which emotion was conveyed by the respective stimulus (neutral, happy, angry, sad, disgusted, other emotion) and which emotional intensity the stimuli were spoken in (low intensity, high intensity, other). Only stimuli that were rated by 10 out of 13 raters to convey the correct emotion in the intended intensity were included in the study.

The oddball paradigm was presented in E-Prime version 1.2 [75] with an interstimulus interval of 600 ms in task 1 (passive task, no response required) and with an interstimulus interval of 1000 ms in task 2 (active task, response required) in order to give participants a sufficient time window for their responses. Each task was initiated by a habituation phase consisting of 20 standards and was presented in a pseudo-randomized manner (different for each participant) with the constraint of two deviants never occurring in succession. The probability of a deviant to occur was the same (20%) in task 1 and 2.

### Procedure

EEG activity was recorded from 64 tin electrodes mounted in an elastic electro cap organized according to the international 10/20 system. EEG data were recorded with a linked mastoid physical reference and were re-referenced by using an average reference. Electrooculogram (EOG) was recorded for artefact rejection purposes from electrodes placed on the supraorbital and ridges of the left eye. The ground electrode was applied to the sternum. Impedance of all electrodes was kept below 5 kΩ for each participant. EEG was continuously recorded with a sampling rate of 500 Hz, amplified, and off-line digitally low-pass filtered with a cut-off frequency of 30 Hz.

Participants were seated in front of a monitor at a distance of approximately 50 cm in a dimly lit, electrically shielded and sound-attenuated cabin. The auditory oddball paradigm was presented via loudspeakers placed at the left and right side of the monitor at approximately 70 dB, while the EEG was recorded.

In task 1, participants watched the first 20 minutes of a silent cartoon movie (title: Kiki's delivery service) and were instructed to focus on the story in the movie while ignoring the sounds. In the oddball paradigm used in task 1, neutral prosody served as standards (960 trials), while happy, angry, sad, and disgusted emotional prosody spoken in low intensity served as deviants (60 trials each). Only stimuli of lower salience (low emotional intensity) were used in this task to prevent participants from directing their attention to the auditory stimuli, which enabled us to measure electrophysiological responses to subtle prosodic changes during unattended processing. The ERP component of interest in this task was the MMN.

In task 2, participants were instructed to press a button as fast as possible whenever they heard an emotion different from the standard emotion (irrespective of intensity). They were asked to look at a fixation point in the center of the screen to prevent eye movements. In this oddball paradigm, sad emotional prosody spoken with low and high intensity represented the standards (960 trials), whereas happy, angry, and disgusted emotional prosody (60 trials each) in both intensities served as deviants. Sad emotional prosody instead of neutral intonation was used as standard in this task because the aim of this task was to record ERP responses to actual changes in the perception of one emotional intonation to another as it frequently occurs in real life, rather than a change from a neutral to an emotional intonation. The total duration of task 2 was 32 minutes, and participants could take a break after the first 16 minutes, if needed. The ERP components of interest in this task were the early components N1 and P2, and the late component P3.

Task 3 was an off-line task, in which participants were given a list to identify both the emotion a nonsense syllable was spoken in as well as the intensity of the emotion. Fifty-four stimuli were presented at an interstimulus rate of five seconds to give participants sufficient time to mark the identified emotion and intensity on the list.

### ERP data analysis

EEG data were analyzed with Brain Vision Analyzer (version 1.05) by means of peak analyses. Prior to averaging, trials with eye-movement and blink artefacts were excluded from analysis. Criteria for artefact rejection were a maximal voltage step of 50 µV, a maximal difference between two values in a segment of 100 µV, and a minimal and maximal amplitude of −100 µV and 100 µV, respectively. All averages were aligned to a 100 ms pre-stimulus baseline. In accordance with the MMN literature, MMN parameters were calculated from a difference waveform obtained by subtracting the standard-stimulus ERPs from the deviant-stimulus ERPs.

For task 1, a total mean number of 229.5 deviant trials (SD 6.6) in emotional prosody were recorded for each of the 60 participants, with a mean number of 57.4 trials for happy, angry, sad, and disgusted prosodic deviants, respectively. Artefact rejection excluded a mean percentage of 12.2 percent of all trials, leaving a total mean of 201.5 (SD 8.8) deviant trials for analysis, with a mean number of 50.4 trials per emotional deviant condition. For task 2, a total mean number of 172.9 (SD 5.8) deviant trials in emotional prosody was recorded, with a mean number of 57.6 trials per emotional deviant condition. Artefact rejection excluded a mean percentage of 10.6 percent of all trials, leaving a total mean of 154.6 deviant trials (SD 8.5) for analysis, with a mean number of 51.5 trials per condition for each of the 60 participants.

Time-windows for peak detection were time-locked to the onset of standards and deviants. The time-windows were chosen in agreement with the existing literature on the respective ERP component and based on visual inspection of the data. The latter revealed that the MMN during unattended processing of emotional prosody consistently exhibited a double-peak across participants. Thus, two MMN peaks were identified in a time-windows of 50–130 ms post-onset for the first peak (in the following referred to as eMMN) and 130–250 ms post-onset for the second peak (in the following referred to as lMMN). Consequently, separate statistical analyses were conducted for amplitudes and latencies of the detected peaks in the eMMN and the lMMN time-windows.

**Figure 1:**
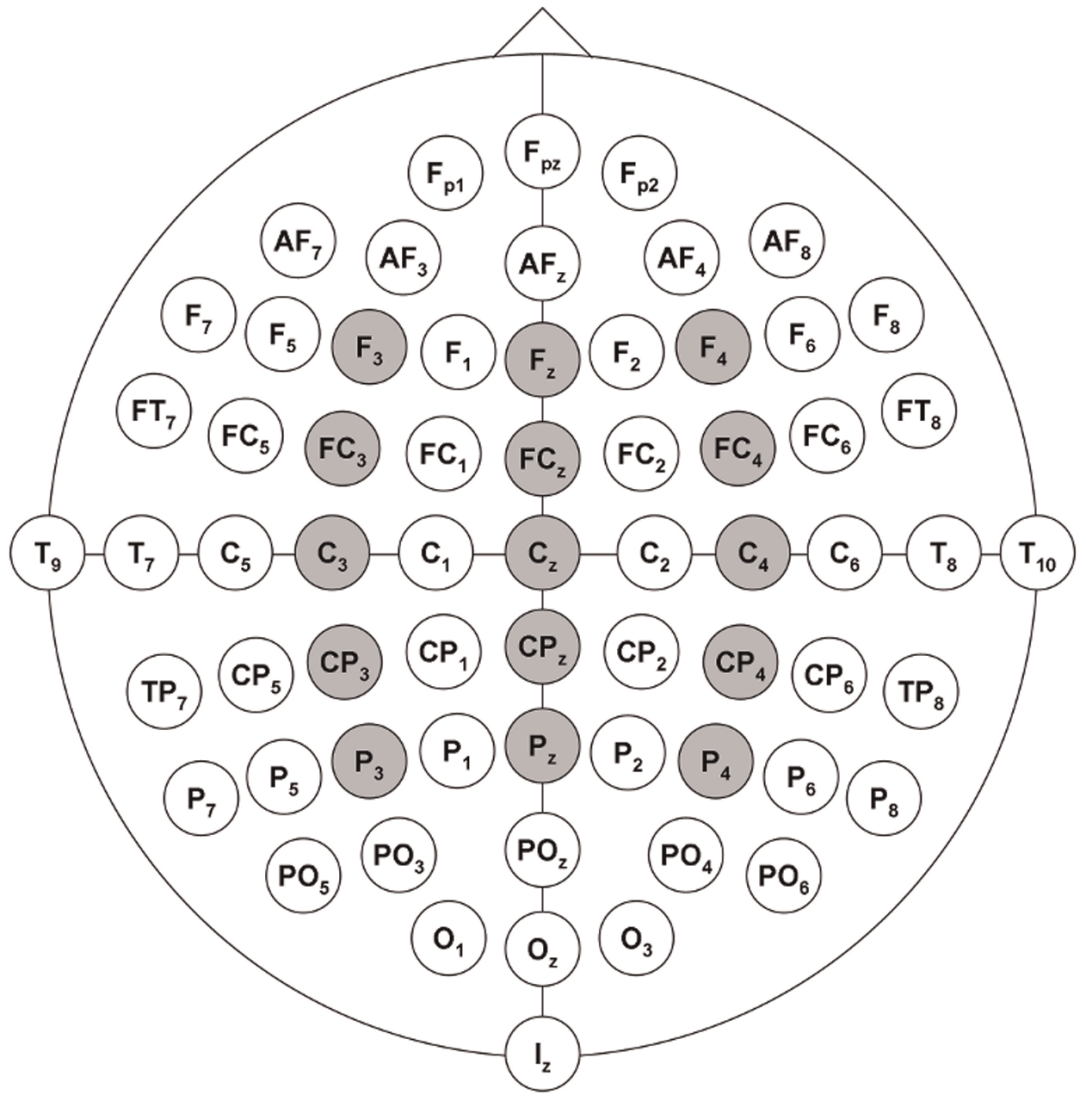
Electrode Map. Electrode map with electrodes used for analysis identified. Factors included in analysis: laterality (left hemisphere, midline, right hemisphere), and region (frontal, frontocentral, central, centroparietal, and parietal).

During attended processing of emotional prosody (task 2), peaks were identified in the following time-windows: 90–140 ms post-onset for the N1, 150–250 ms post-onset for the P2, and 310–550 ms post-onset for the P3. Because the P3 does not always exhibit a clear peak, results of the peak detection procedure were inspected in each subject and trials that did not show a clear peak in the defined time-window (due to multiple peaks) were excluded. Statistical analyses were conducted for amplitudes and latencies of the detected peaks in the N1, P2, and P3 time-windows. In order to obtain symmetrical coverage of the scalp during statistical analysis, five midline electrodes were chosen covering frontal through parietal areas (Fz, FCz, Cz, CPz, Pz) along with their corresponding left (F3, FC3, C3, CP3 and P3) and right (F4, FC4, C4, CP4, P4) counterparts (see figure 1).

As the results of task 1 show, alexithymia scores were found to modulate ERP responses to sad prosody, which served as standards in task 2. To exclude the possibility that differences in ERP responses to sad standards confounded ERP responses to deviants in task 2, peak amplitudes in response to deviants were subtracted by peak amplitudes in response to standards taking intensity difference into account (i.e. low intensity standards were subtracted from low intensity deviants, high intensity standards were subtracted from high intensity deviants). This procedure ensured that voltage changes elicited by deviants were measured relative to voltage changes elicited by standards. The procedure further led to better comparability of the results of task 1 and 2, as in both tasks standard-related activity was subtracted from activity elicited by deviants and the resulting difference waves were used in subsequent statistical analyses.

### Statistical data analysis

#### Behavioral data

Statistical analyses were performed in SPSS version 17.0 (SPSS Inc; Chicago, Illinois). Behavioral data were available from 57 (out of 60) subjects. Two subjects did not complete the behavioral task due to time restrictions as a consequence of technical difficulties at the beginning of the session. One subject with an outlier score (67) on the affective dimension of alexithymia was excluded to ensure continuity of scores. Scores of the remaining 57 subjects ranged from 20 to 55 on the affective alexithymia dimension (median: 35, SD: 9.7) and from 27 to 93 on the cognitive alexithymia dimension (median: 52, SD: 17.1). Pearson's correlations were performed between each alexithymia dimension and error rates as well as RT during prosodic deviant detection (Task 2), and with error rates during emotional prosody identification (Task 3).

#### ERP data

During unattended processing of emotional prosody (task 1), eMMN and lMMN peak amplitudes and latencies elicited by happy, angry, sad, and disgusted prosodic deviants were analyzed. From the 60 subjects, the data of four subjects had to be discarded due to large amounts of eye blink and motion artefacts leaving an insufficient number of target trials for analysis. Four (emotion: happy vs. angry vs. sad vs. disgusted) by 3 (laterality: left hemisphere vs. midline vs. right hemisphere) by 5 (region: frontal vs. frontocentral vs. central vs. centroparietal vs. parietal) repeated-measures multivariate analyses of covariance (RM-MANCOVA) were then performed on the data of the remaining 56 participants with scores on the affective and cognitive alexithymia dimensions included as covariates. In case of significant interactions of factors with the covariates, follow up MANCOVAs were conducted for each level of the respective factor in order to identify the sources of the effect. In addition, MANCOVAs including each subscale of the respective alexithymia dimension as covariates were conducted in order to test which subscale significantly contributed to the effect.

During attended processing of emotional prosody (task 2), peak amplitudes and latencies of the N1, P2, and P3 elicited by happy, angry, and disgusted prosodic deviants spoken in low (e.g. a bit angry) and high (e.g., very angry) intensity were analyzed in 57 subjects. The data of three subjects were discarded due to high amounts of eye blink and motion artefacts. Only trials of correctly detected prosodic deviants were included in the analysis. Affective scores of subjects in task 2 ranged from 20 to 55 (median: 35, SD: 9.7), cognitive scores ranged from 27 to 93 (median: 53, SD: 17.4). Two (intensity: low vs. high) by 3 (emotion: happy vs. angry vs. disgusted) by 3 (laterality: left vs. middle vs. right) by 3 (region) RM-MANCOVAs were conducted with affective and cognitive alexithymia scores included as covariates. Follow up MANCOVAs were conducted to identify the sources and the contribution of separate BVAQ subscales to the observed effects.

The three levels of the factor region varied for the N1, P2 and P3 in accordance with the known topographic distributions of these components when elicited in the auditory modality: The N1 has a central maximum and was therefore analyzed at frontocentral, central, and centroparietal regions. The P2 is maximal over anterior regions, the analysis therefore comprised anterior electrode sites (frontal, frontocentral, and central). The P3 is known to have a centroparietal-parietal maximum, thus central, centroparietal, and parietal regions were included in the P3 analysis.

In case of sphericity violations, Greenhouse-Geisser corrected p-values are reported. A Sidak correction of p-values was used in pairwise comparisons between the levels of factors. Results are reported with a focus on main effects and interactions with the affective and cognitive alexithymia dimensions.

## Results

### Behavioral data

Pearson's correlation confirmed a high correlation between the cognitive dimension of alexithymia as assessed by the three cognitive BVAQ subscales and the TAS-20 total score (*r* = 0.85, *p*<0.01).

Behavioral data of task 2 (detection of prosodic deviants) revealed no significant correlations of the cognitive and affective alexithymia dimensions with accuracy (ACC, cognitive dimension: r = .092, p = .496, affective dimension: r = .024, p = .861) and reaction time (RT, cognitive dimension: r = −.174, p = .197, affective dimension: r = −.093, p = .489).

**Figure 2:**
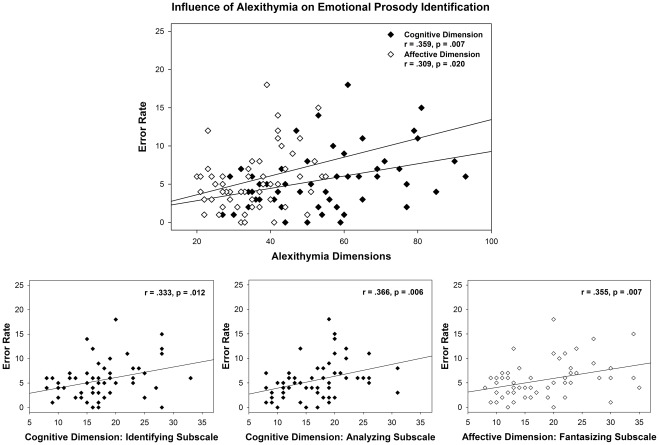
Behavioral Results: Identification of Emotional Prosody. Behavioral results of emotional prosody identification (task 3). Top panel: Correlation between the affective and the cognitive alexithymia dimension with error rates. Bottom panel: Correlations of the cognitive subscales ‘difficulty identifying feelings’ (left), ‘difficulty analyzing feelings’ (middle) and of the fantasizing subscale of the affective alexithymia dimension (right) with error rates.

As shown in figure 2, both alexithymia dimensions correlated significantly with error rates during the more difficult identification of emotional prosody in task 3 (affective dimension: r = .309, p = .020, cognitive dimension: r = .359, p = .007), which were found to result from increased error rates during the identification of disgusted prosody only. Correlations with the separate BVAQ subscales showed that the correlation between the affective dimension and error rates was driven only by the fantasizing subscale (r = .355, p = .007), while the correlation between the cognitive dimension and error rates was driven by all three cognitive subscales (identifying: r = .333, p = .012, verbalizing: r = .266, p = .048, analyzing: r = .366, p = .006). Error rates on the identification of emotional intensity were unrelated to either alexithymia dimension (p>.05).

In summary, both alexithymia dimensions were associated with significantly worse performance on the identification of disgusted prosody, while performance on emotional intensity identification was unrelated to alexithymia.

### ERP data: Unattended processing of emotional prosody

**Figure 3:**
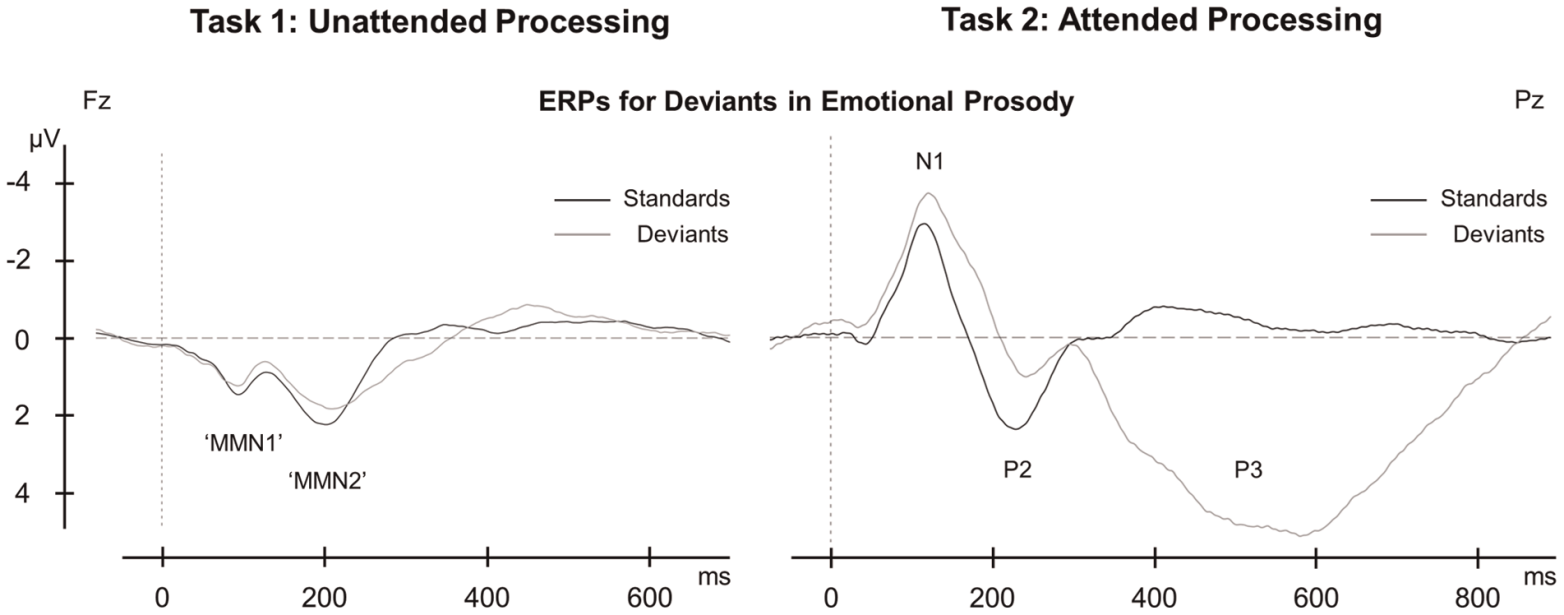
ERP Results: MMN and P3 for Emotional Prosody in Alexithymia. ERP results for task 1 (left, unattended processing) and task 2 (right, attended processing). Grand averages of all subjects at electrode site Fz for the eMMN and lMMN and at electrode site Pz for the P3, corresponding to the topographic distribution of these components. Standards versus deviants, with ERP components used for analysis identified. ‘EMMN’ and ‘lMMN’: depicted are the actual ERPs elicited by deviants in emotional prosody. For statistical analysis, difference waves were calculated for the eMMN and lMMN in correspondence with the common procedure by subtracting ERPs elicited by standards from ERPs elicited by deviants.

Figure 3 (left panel) shows the eMMN and lMMN elicited by deviants in emotional prosody (happy, angry, sad, and disgusted deviants averaged) versus neutral standards during unattended processing in task 1 (grand average across all subjects at the frontal electrode site Fz). All main effects and interactions for amplitudes and latencies of the eMMN and lMMN are summarized in table 1 (affective alexithymia dimension) and table 2 (cognitive alexithymia dimension).

**Table 1:** Statistical results of the RM-MANCOVA including the affective alexithymia dimension as a covariate.

	Main effects and interactions	Post hoc tests
**EMMN**		
Amplitude	Region *F* (4,216) = 10.892, *p*<.001 Laterality × Region *F* (8,432) = 5.751, *p*<.001	
Latency	Region *F* (4,216) = 4.521, *p* = .014	
**LMMN**		
Amplitude	Region *F* (4,216) = 4.990, *p* = .019 Laterality × Region *F* (8,432) = 2.778, *p* = .030	
Latency	Emotion *F* (3,162) = 4.565, *p* = .004 Emotion × Region *F* (12,648) = 2.269, *p* = .046	
**N1**		
Amplitude	No significant effects	
Latency	No significant effects	
**P2**		
Amplitude	Region *F* (2,114) = 22.065, *p*<.001 Region × Affective Dimension *F* (2,114) = 6.836, p = .006	Reduced P2 peak amplitudes at frontal regions with increasing scores on the affective alexithymia dimension (fantasizing subscale)
Latency	Laterality *F* (2,102) = 3.406, *p* = .037	
**P3**		
Amplitude	Intensity *F* (1,56) = 6.044, *p* = .017 Intensity × Affective Dimension *F* (1,56) = 5.792, p = .019 Laterality *F* (2,112) = 7.322, *p* = .001 Intensity × Region *F* (2,112) = 4.459, *p* = .037 Intensity × Region × Affective Dimension *F* (2,112) = 4.093, *p* = .046	Stronger reduction in P3 peak amplitudes for high intensity prosody compared to low intensity prosody with increasing scores on the affective alexithymia dimension (emotionalizing subscale) Reduced P3 peak amplitudes at central regions for high intensity prosody with increasing scores on the affective alexithymia dimension (fantasizing subscale)
Latency	Region *F* (2,112) = 4.964, *p* = .017	

Post hoc tests are significant at p<.05 (Sidak-corrected) unless otherwise specified.

#### EMMN

RM-MANCOVA showed that peak amplitudes of the eMMN did not differ as a function of alexithymia. EMMN peak latency, however, was associated with the cognitive, but not the affective alexithymia dimension, as a significant interaction *cognitive dimension* × *laterality* revealed [*F*(2,108) = 4.160, p = .018], suggesting that the eMMN peaked significantly earlier in the left hemisphere and tended to peak later in the right hemisphere with increasing scores on the cognitive alexithymia dimension. Follow up tests on each cognitive subscale showed that this interaction was driven by *difficulty verbalizing feelings* [*F*(2,108) = 3.852, p = .024] and *difficulty identifying feelings* [*F*(2,108) = 34.232, p = .017]. In addition, *difficulty identifying feelings* interacted with *emotion* [*F*(3,126) = 3.828, p = .011], indicating that for disgusted prosody only, eMMN peak latencies were delayed with increasing *difficulty identifying feelings*.

#### LMMN

Peak latencies and amplitudes of the lMMN did not differ as a function of either alexithymia dimension, suggesting that alexithymia did not affect the global processing of unattended changes in emotional speech.

In summary, difficulty verbalizing and identifying feelings as part of the cognitive alexithymia dimension were associated with an earlier left-hemispheric response and a trend toward a delayed right-hemispheric response during the early acoustic encoding (eMMN) of subconsciously perceived variations in emotional speech. Difficulty identifying feelings was further associated with delayed encoding of disgusted prosody. In contrast, the affective alexithymia dimension was not associated with altered unattended processing of emotional speech. Neither alexithymia dimension affected the later stage of global acoustic processing (lMMN).

### ERP data: Attended processing of emotional prosody

Figure 3 (right panel) shows the N1, P2, and P3 elicited by deviants in emotional prosody (happy, angry, and disgusted deviants averaged) versus sad standards during attended processing in task 2 (grand average across all subjects at the parietal electrode site Pz). All main effects and interactions for amplitudes and latencies of the N1, P2, and P3 are summarized in table 1 (affective alexithymia dimension) and table 2 (cognitive alexithymia dimension).

**Table 2:** Statistical results of the RM-MANCOVA including the cognitive alexithymia dimension as a covariate.

	Main effects and interactions	Post hoc tests
**EMMN**		
Amplitude	Region *F* (4,216) = 8.327, *p* = .002 Laterality × Region *F* (8,432) = 4.554, *p* = .002	
Latency	Laterality *F* (2,108) = 4.616, *p* = .012 Region *F* (4,216) = 6.341, *p* = .003 Laterality × Cognitive Dimension *F* (2,108) = 4.160, *p* = .018	Earlier left-hemispheric and trend toward delayed right-hemispheric eMMN peak amplitude (p<.1) with increasing scores on the cognitive alexithymia dimension (verbalizing and identifying subscales)
**LMMN**		
Amplitude	Laterality × Region *F* (8,432) = 3.696, *p* = .007	
Latency	Emotion *F* (3,162) = 4.775, *p* = .003 Laterality *F* (2,108) = 4.338, *p* = .015	
**N1**		
Amplitude	Region *F* (2,114) = 4.304, *p* = .037 Region × Cognitive Dimension *F* (2,114) = 4.335, p = .037	Enhanced N1 peak amplitudes at centroparietal regions with increasing scores on the cognitive alexithymia dimension (analyzing subscale)
Latency	No significant effects	
**P2**		
Amplitude	Region *F* (2,114) = 17.738, *p*<.001 Laterality × Cognitive Dimension *F* (2,114) = 4.128, *p* = .037	Reduced P2 peak amplitudes at frontal regions with increasing scores on the cognitive alexithymia dimension (analyzing subscale)
Latency	Laterality *F* (2,114) = 8.897, *p*<.001	
**P3**		
Amplitude	Emotion *F* (2,112) = 3.378, *p* = .038 Laterality *F* (2,112) = 8.013, *p* = .001 Region *F* (2,112) = 21.881, *p*<.001 Region × Cognitive Dimension *F* (2,112) = 9.125, p = .003	Reduced P3 peak amplitudes at parietal regions with increasing scores on the cognitive alexithymia dimension (verbalizing and identifying subscale)
Latency	Region *F* (2,112) = 14.277, *p*<.001	

Post hoc tests are significant at p<.05 (Sidak-corrected) unless otherwise specified.

#### N1

For the cognitive dimension, a significant interaction *cognitive dimension* × *region* [*F*(2,114)  = 4.335, *p* = .037] was found for peak amplitudes of the N1, suggesting larger N1 amplitudes at centroparietal regions with increasing scores on the cognitive alexithymia dimension. Follow up tests on each cognitive subscale revealed that this interaction was driven only by *difficulty analyzing feelings* [*F*(2,114) = 4.677, *p* = .030]. Peak latencies of the N1 showed no difference as a function of the cognitive alexithymia dimension. The affective dimension was not associated with differences in N1 amplitude or latency.

#### P2

For the cognitive dimension, RM-MANCOVA on P2 peak amplitudes revealed a significant interaction *cognitive dimension* × *region* [*F*(2,114)  = 4.128, *p* = .037]. Follow up tests showed that this interaction was driven only by the subscale *difficulty analyzing feelings*, which was associated with reduced frontal P2 amplitudes.

**Figure 4:**
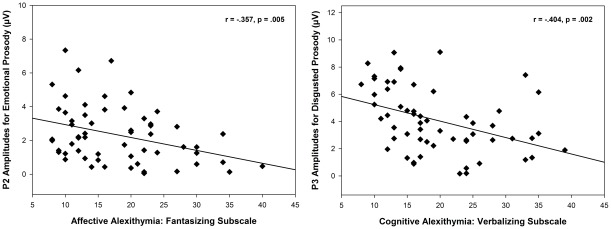
Correlations of Alexithymia Dimensions with P2 and P3 Amplitudes. Left: Negative correlation between the affective alexithymia dimension (fantasizing subscale) with P2 amplitudes in response to deviants in emotional prosody. Right: Negative correlation between the cognitive alexithymia dimension (verbalizing subscale) with P3 amplitudes in response to disgusted prosodic deviants.

For the affective dimension, there was also a significant interaction *affective dimension* × *region* [*F*(2,114)  = 6.836, *p* = .006]. Follow up tests revealed that this interaction was driven by the *fantasizing* subscale [*F*(2,114)  = 6.077, *p* = .010], which was found to be associated with reduced frontal P2 amplitudes (Figure 4, left).

Peak latency of the P2 did not vary as a function of either alexithymia dimension.

#### P3

For the cognitive dimension, RM-MANCOVA showed a significant interaction *cognitive dimension* × *region* [*F*(2,112)  = 9.125, p = .003], suggesting reduced amplitudes of the P3 at parietal regions with increasing scores on the cognitive alexithymia dimension. Follow up tests on each subscale revealed that this interaction was driven by *difficulty verbalizing feelings* [*F*(2,112)  = 12.544, p<.001] and *difficulty identifying feelings* [*F*(2,112)  = 5.711, p = .017], which were both associated with reduced P3 amplitudes at parietal regions. In addition, the *verbalizing* subscale showed a significant three-way interaction *verbalizing* × *emotion* × *region* [*F*(4,224)  = 3.087, p = .046]. Follow up tests on each emotion revealed a significant interaction *verbalizing* × *region* [*F*(2,112)  = 7.946, p = .001] for disgusted prosody, indicating that *difficulty verbalizing feelings* was associated with reduced parietal P3 amplitudes particularly during the detection of disgusted prosodic deviants (Figure 4, right).

For the affective dimension, RM-MANCOVA showed a significant *affective dimension* × *intensity* interaction [*F*(1,56)  = 5.792, p = .019], which was further qualified by the factor *region* [*affective dimension* × *intensity* × *region*: *F*(2,112)  = 4.093, p = .046]. Follow up tests on each affective subscale revealed significant effects of both the *emotionalizing* and the *fantasizing* subscale: For the *emotionalizing* subscale, there was a significant interaction *emotionalizing* × *intensity* [*F*(1,56)  = 6.433, p = .014], suggesting that the reduction in P3 amplitudes with increasing scores on *emotionalizing* was even more pronounced for deviants in emotional prosody spoken with high intensity than for those spoken with low intensity. For the *fantasizing* subscale, there was a three-way interaction *fantasizing* × *intensity* × *region*: *F*(2,112)  = 6.414, p = .013]. Follow up tests on the factor *region* revealed a significant interaction *fantasizing* × *intensity* [*F*(1,56)  = 6.455, p = .014] at central regions, suggesting a reduction in central P3 amplitudes with increasing scores on *fantasizing* only for high intensity deviants in emotional prosody.

#### Summary of ERP data

In summary, electrophysiological data for the detection of changes in emotional prosody suggested that alexithymia was significantly associated with alterations of early (N1, P2) as well as late (P3) ERP components. A dissociable impact of the two dimensions of alexithymia was observed: Only the cognitive dimension (analyzing subscale) was related to larger amplitudes of the early N1 component, while both the cognitive (analyzing subscale) and the affective dimensions (fantasizing subscale) were associated with reduced frontal P2 amplitudes. Both alexithymia dimensions were associated with reduced amplitudes of the late P3 component, but in a qualitatively different fashion: Individuals with difficulty verbalizing and identifying feelings (cognitive dimension) showed similarly reduced P3 amplitudes at parietal regions for all deviants in emotional prosody irrespective of the specific emotion and intensity, with the exception that individuals with difficulty verbalizing feelings exhibited a particularly diminished P3 response to disgusted prosody. In contrast to the cognitive dimension, the affective alexithymia dimension was sensitive to the difference in intensity with which the prosodic deviants were pronounced: High scores on emotionalizing (i.e., low emotional arousal) were associated with even stronger P3 amplitude reductions for prosodic deviants spoken with high intensity compared to those spoken with low intensity, while high scores on fantasizing (i.e. low abilities to fantasize, imagine etc.) were associated with reduced P3 amplitudes only for prosodic deviants spoken with high emotional intensity.

Control analyses of all behavioral and electrophysiological data using the TAS-20 showed highly comparable results with the cognitive alexithymia dimension assessed with the BVAQ.

## Discussion

The present study investigated the impact of the cognitive and affective alexithymia dimensions on the electrophysiological processing of attended and unattended emotional prosody. At unattended processing levels, the cognitive dimension was associated with a left-hemisphere bias during the early acoustic encoding of emotional speech, as reflected in overall shorter left-hemispheric eMMN latencies. No effect of the affective dimension was observed during the unattended processing of emotional speech. At attended processing levels, the cognitive alexithymia dimension modulated both early and late ERP components, reflected in larger N1 amplitudes and reduced P2 and P3 amplitudes. In contrast, the affective dimension did not modulate the early N1 component, but was also linked to reduced P2 amplitudes, and further showed a negative association with P3 amplitudes particularly for emotional prosody spoken with high emotional intensity. These results suggest that alexithymia modulates electrophysiological responses to emotional speech at attended as well as unattended processing levels, and provide evidence for a dissociable impact of the cognitive versus the affective alexithymia dimension on the processing of the emotional qualities of speech.

### Behavioral performance

Behavioral data of the present study show that the mere detection of deviants in emotional prosody (task 2) was not affected by either alexithymia dimension. This is in line with the two previous studies on the relation between alexithymia and emotional prosody, which did not observe behavioral differences as a function of alexithymia during emotional prosody identification at the sentence level [15] and during cross-modal affective priming [44]. However, our findings indicate that when participants are asked to specifically identify the emotion conveyed by brief vocal stimuli (task 3), deficits did become apparent, as evidenced by worse performance on disgusted prosody identification with increasing scores on both alexithymia dimensions. This could explain why behavioral differences were not observed in our previous study [44], as no disgusted but only happy and sad prosody were employed. Moreover, the current study asked subjects to identify the emotional prosody of brief vocal stimuli (600 ms), whereas the previous behavioral study on emotional prosody identification [15] employed full sentences with much longer duration, giving participants more time to identify the conveyed emotion. Thus, lower task difficulty in the latter study [15] could explain why alexithymia-related differences were not detected at the behavioral level.

Taken together, our behavioral findings are in line with previous reports of difficulty in the identification of visually displayed emotion in alexithymia [13]–[15] and indicate that such emotion identification problems extend to the auditory domain. However, our finding of worse performance only during disgusted prosody identification points toward a rather subtle deficit in emotional prosody identification. This seems not surprising considering that individuals scoring high on alexithymia are, despite their interpersonal problems, generally high-functioning, socially adapted individuals. The pursuit of social conformity is a characteristic feature of alexithymia [2] and implies learning to interpret emotional signals during social communication to the best of one's ability.

### Unattended processing of emotional prosody

Alexithymia was found to affect amplitudes of the eMMN during unattended processing of emotional prosody (task 1). We observed a left-hemisphere bias during early acoustic encoding (as indexed by a faster left-hemispheric eMMN) with increasing scores on the identifying and verbalizing subscale of the cognitive alexithymia dimension for all deviants in emotional prosody (happy, angry, sad, and disgusted), which was additionally paired with a tendency toward a delayed response of the right hemisphere. Difficulty identifying feelings was further associated with overall delayed eMMN latencies to disgusted prosody.

These findings are particularly interesting considering that amplitudes of the neuromagnetic equivalent of the MMN in response to changes in emotional prosody have recently been found to be larger in the right hemisphere in healthy individuals [76], and that the right hemisphere has long been assumed to play an important role in processing emotional prosody [77], [78]. However, the question of right hemisphere predominance for emotional aspects of speech is still under debate and may constitute a relative rather than an absolute dominance [43], [79], [80]. In any case, our finding of a left-hemisphere bias during early acoustic processing of emotional prosody in cognitive alexithymia is in line with the hypothesis of a hyperactive left hemisphere during emotional processing in this personality trait [32], and suggests that decreased abilities in identifying and verbalizing one's feelings are linked to a hyper-reliance on the left hemisphere, normally specialized for cognitive analyses rather than emotional processing [81].

### Attended processing of emotional prosody

#### Early processing

During attended processing of emotional speech (task 2), the two alexithymia dimensions modulated early (<300 ms) electrophysiological responses to emotional speech in a qualitatively different fashion. Cognitive alexithymia was associated with larger N1 amplitudes in response to detected deviants, an association that was driven only by the subscale difficulty analyzing feelings. Larger N1 amplitudes were also reported in a previous study on auditory emotion perception in alexithymia in response to aversive white noise [42]. Given that the N1 reflects the extraction of acoustic cues during early acoustic processing and that its amplitude increases with attention [48], this may suggest that individuals with difficulty analyzing feelings need to devote more attention to acoustic cues in order to detect changes in emotional prosody. Difficulty analyzing feelings was further associated with reduced frontal P2 amplitudes. The affective alexithymia dimension did not modulate the N1 but was also associated with reduced frontal P2 amplitudes, an association driven only by the fantasizing subscale. The P2 has previously been shown to be sensitive to the emotional qualities of speech and is thought to reflect initial emotional salience detection [50], [51]. Our finding of reduced frontal P2 amplitudes in individuals with difficulty analyzing feelings (cognitive dimension) and impaired abilities to fantasize (affective dimension) could thus represent a reduced initial detection of emotional speech salience, i.e. attenuated basic emotional processing as previously suggested by Pollatos and Gramann [38].

Taken together, these results suggest that alexithymia modulates both the unattended as well as the early attended processing of emotional prosody, with a qualitatively different impact of the two alexithymia dimensions: Only the cognitive dimension was associated with a left hemisphere preference during the unattended acoustic encoding of variations in emotional speech, and with a higher amount of attention devoted to the extraction of acoustic cues when detecting emotional prosodic deviants. An effect of the affective dimension was only found starting at approximately 150 ms (P2 time-window) reflected in reduced frontal P2 amplitudes, which were also observed in relation to the cognitive dimension. These results confirm previous reports of early perceptual differences in emotional processing as a function of alexithymia [35], [38], and extend these findings by suggesting that such early perceptual modulations may be predominantly associated with the cognitive alexithymia dimension, whereas its affective dimension may be related to differences at later stages of emotional appraisal.

In addition, our observation of a faster left-hemispheric response and a tendency toward a delayed right-hemispheric response during the early acoustic encoding of unattended emotional speech is in line with the hypotheses of a left hemisphere preference during emotional processing [26], [32] and a hypoactive right hemisphere associated with alexithymia [30], [82]. However, according to our findings such a left-hemisphere bias and hemispheric dissociation seems predominantly evident in the cognitive dimension of alexithymia. It would be worthwhile to further investigate in future studies whether a left-hemisphere preference and right hemisphere hypoactivity during emotional processing may be a characteristic of particularly the cognitive alexithymia dimension.

#### Late processing

Our results further demonstrated that alexithymia is associated with reduced amplitudes of the later (>300 ms) occurring P3, a component reflecting conscious stimulus evaluation. Amplitudes of this component have been shown to be sensitive to the ascribed importance to a stimulus (the higher the subjective importance, the higher the P3 amplitude). The P3 is also related to the emotional valence assigned to a stimulus in such a way that higher emotional valence is reflected in larger P3 amplitudes [83], [84]. Our finding of reduced P3 amplitudes in response to emotional prosody corroborates and extends the findings of previous studies, which reported reductions in P3 amplitudes during emotional picture processing in alexithymia [36], [38].

In addition, our results indicate differential effects of the two alexithymia dimensions on P3 amplitudes. The cognitive dimension was related to generally smaller P3 amplitudes in response to changes in emotional prosody, an association driven by the subscales difficulty identifying and verbalizing feelings. Difficulty verbalizing feelings was additionally related to reduced P3 amplitudes particularly to disgusted prosody. In contrast, the affective dimension was found to be sensitive to the intensity with which emotional speech was pronounced, reflected in even stronger reductions of P3 amplitudes for intonations spoken in high emotional intensity. This may suggest that individuals with difficulty identifying and verbalizing feelings generally ascribe less significance to emotional speech qualities, a process that may be particularly prominent in the case of disgusted prosody. Regarding the affective alexithymia dimension, this process might be even more pronounced for emotional prosody spoken with high intensity.

#### A specific role for disgusted prosody

Though all prosodic emotions tested in the present study were affected by alexithymia, we observed some indications for a specific role of disgusted prosody. Individuals with difficulty identifying feelings as part of the cognitive alexithymia dimension exhibited overall delayed eMMN peak latencies for disgusted prosody, suggesting a delay in the unattended acoustic encoding of disgust conveyed by speech. Further, individuals with difficulty verbalizing feelings showed particularly reduced P3 amplitudes in response to the detection of disgusted prosodic deviants. Though no special role of disgust at the electrophysiological level was found in relation to the affective alexithymia dimension, both dimensions were associated with impaired identification of disgusted prosody at the behavioral level. These results may indicate a specific role of disgusted prosody during emotional speech processing in alexithymia. However, previous findings on facial emotion recognition do not seem to suggest a specific role of disgusted emotion, but indicate a more general deficit in emotion recognition associated with alexithymia [82], [85], [86]. As the auditory perception of disgust has not been investigated before in relation to alexithymia, it may be worthwhile to test the possibility of a specific deficit in the processing of disgusted prosody in future studies.

### Summary

In summary, the present findings hint toward a dissociable impact of the cognitive and affective alexithymia dimensions on the processing of emotional speech qualities. These results provide further support to the notion that the two alexithymia dimensions may be differentially linked to emotional processing [9], [10]. Based on the distinction between these two dimensions, the existence of two different subtypes of alexithymia has been proposed [8], [87]. Individuals with type I alexithymia are thought to be characterized by a general lack of responsiveness to emotion at both the cognitive level and the level of emotional experience, whereas individuals with type II alexithymia experience feelings to a normal or even heightened degree, whereas their ability to cognitively regulate their feelings is impaired, possibly putting them at risk to develop psychopathological conditions characterized by affect dysregulation [10]. Future studies could attempt to differentiate between different alexithymia subtypes taking into account the affective alexithymia dimension in addition to its cognitive dimension, rather than considering alexithymia as a unitary construct. Such a differentiation would be beneficial to a better understanding of the neurophysiological basis of emotional processing deficits associated with this multifaceted personality construct.

### Limitations

It should be kept in mind that the sample of the present study comprised only female participants, and that our results may therefore not be generalizable to male individuals with alexithymia. Also, mood states of participants were not assessed though alexithymia has been associated with reduced positive affect [88]. Further, the range of affective alexithymia scores in our sample was relatively small compared to the range of scores on the cognitive alexithymia dimension. Future studies should try to overcome these limitations by testing a sufficient number of female and male individuals with a broad range of scores on both the affective and the cognitive dimension of alexithymia. In addition, we used sad prosody as standards during attended processing in task 2 as we were interested in measuring changes between emotional intonations as they often occur in daily conversations instead of changes from a neutral to an emotional intonation. However, since neutral prosody served as standards during unattended processing in task 1, it should be noted that the results of the two tasks are not directly comparable. Future studies could attempt to keep paradigms and stimuli identical during unattended and attended processing if specific effects of attention on emotional prosody processing in relation to alexithymia are of interest.

### Conclusions

In conclusion, alexithymia seems to modulate electrophysiological responses to emotional speech during attended as well as unattended processing. The two alexithymia dimensions appear to exert a dissociable impact on emotional prosody processing, with a left-hemisphere bias characteristic for the cognitive alexithymia dimension during early stages of unattended processing. The affective alexithymia dimension seems to influence the perception of emotional prosody at later processing stages than the cognitive dimension, and appears to be additionally sensitive to the intensity of emotional speech. These results suggest that alexithymia indeed affects the way emotional speech qualities are processed in the brain, which could be a contributing factor to problems in interpersonal communication associated with this personality construct.
